# Growth Performance and Gut Health of Cold-Stressed Broilers in Response to Supplementation with a Combination of Sodium Butyrate and Vitamin D3

**DOI:** 10.3390/ani15060861

**Published:** 2025-03-17

**Authors:** Hang Gao, Yi Wang, Xingkai Zhao, Yaling Yu, Yizhe Guo, Zhendong Li, Zhenlei Zhou

**Affiliations:** 1 College of Veterinary Medicine, Southwest University, Chongqing 400715, China; vetgaoh@163.com (H.G.);; 2 College of Veterinary Medicine, Nanjing Agricultural University, Nanjing 210095, China

**Keywords:** broiler growth, intestinal health, broiler nutrition, broiler feed additives

## Abstract

The current experiment aimed to investigate the effects of sodium butyrate (SB) and vitamin D3 (VD3) supplementation on the growth performance, immune status, antioxidant capacity, and gut health of young broilers under cold stress. A total of 144 1-day-old Arbor Acres chicks were randomly allotted to three treatments with 6 replicates of 8 birds: (1) basal diet; (2) basal diet + cold stress; and (3) basal diet with 1 g/kg SB and 2000 IU/kg VD3 + cold stress. Birds were exposed to cold stress at 16 ± 1 °C for 72 h (d 18–21) and 26 ± 1 °C for the control. The results indicated that the SB/VD3 diet could alleviate the reduction in average daily gain (ADG) caused by cold stress (*p* < 0.05). The SB/VD3 diet decreased the serum endotoxin level and ileal interleukin-1β gene expression and upregulated interleukin-10 and nuclear factor erythroid 2-related factor 2 (Nrf2) gene expression compared with cold-stressed birds (*p* < 0.05). Furthermore, cold stress altered the composition of gut microbiota, including a decrease in *Clostridium_sensu_stricto_1*, whereas the SB/VD3 diet prevented the reduction. In conclusion, the SB/VD3 diet mitigated the negative effects of cold stress on growth performance and the intestines by strengthening intestinal barrier function and stabilizing gut microbiota balance in broiler chicks, and these results can help to manage cold stress.

## Simple Summary

Cold stress is an environmental stressor that severely threatens growth and intestinal homeostasis in broiler chicks. The current experiment aimed to investigate the effects of combined supplementation with sodium butyrate and vitamin D3 on the growth performance and gut health of young broilers under cold stress. The results indicated that sodium butyrate and vitamin D3 diet could alleviate the reduction of ADG, decrease the serum endotoxin level and ileal IL-1β gene expression, upregulate IL-10 and Nrf2 gene expression, and regulate the composition of the gut microbiota compared with cold-stressed birds. In conclusion, the sodium butyrate and vitamin D3 diet mitigated the negative effects of cold stress on growth performance and the intestines by strengthening intestinal barrier function and regulating gut microbiota balance in broiler chicks. The findings of these results provide an effective dietary strategy for combating cold stress in broiler farming.

## 1. Introduction

Dramatic changes in ambient temperatures are one of the major threats to the sustainability of the global poultry industry [1]. As the world’s most consumed source of animal protein, newly hatched broiler chicks are susceptible to cold stress caused by extreme weather due to their immature thermoregulatory mechanisms and pre-feathering stage. Upon exposure to cold stress, chicks allocate more energy from feed to protect themselves from the cold, resulting in higher basal metabolic rates and feed conversion rates [2]. In addition to its negative effects on growth performance, cold stress impairs the development and function of the gut [3] and even leads to pathological changes. For instance, it was observed that when chicks experienced acute cold stress, intestinal histopathologic analyses showed shortened intestinal villi, mucosal hemorrhage, and inflammatory cell infiltration [4]. In addition, cold stress increases susceptibility to intestinal diseases such as *E. coli* disease [5] and necrotizing enterocolitis [6]. Intestinal homeostasis is critical for maintaining stable growth in commercial broilers. Several dietary strategies, such as feed additives or feed ingredients including probiotics, prebiotics [7], amino acids [8], and antioxidants [9] are often applied in combination to counteract the effect of cold stress on the growth and gut of broilers. Feed compositions play an important role in modulating the biological response of birds to adverse events, as they are the source of energy and metabolites required for all cellular and systemic processes and determine immune response and growth steadiness [10]. Therefore, there is an urgent need for poultry researchers to develop more nutritional dietary strategies to improve cold tolerance and maintain gut homeostasis to mitigate the negative effects of cold stress.

Butyrate is one of the most abundant metabolites produced by microbial fermentation from undigested dietary carbohydrates and has been recognized as an important mediator in the regulation of whole-body energy balance by the gut microbiota [11]. Butyrate, especially sodium butyrate (SB), has been widely used in the poultry industry as an alternative to antibiotic growth promoters [12]. SB improved the growth performance of chicks by promoting intestinal morphological parameters and the expression of avian β-defensin genes [13]. In a long-term supplementation trial, microencapsulated SB was found to dynamically alter the composition of the broiler gut microbiota in a direction that favored host health [14]. Maintaining a balanced gut microbiota is crucial for optimizing the growth performance and immune status of broilers [15] and has emerged as one of the key strategies in promoting gut health [16]. Dietary SB improves female broiler breeder performance and offspring immune function through the gut microbiota [17] and even mitigates *Clostridium perfringens*-induced intestinal villus loss and shortening [18]. In addition, it has been shown that cold exposure directly increases cecal butyrate concentrations, counteracting the cold environment by promoting fat thermogenesis [19].

Vitamin D3 (VD3), an essential micronutrient in the broiler diet, plays a classical role in regulating calcium and phosphorus metabolism to maintain bone mineral homeostasis [20,21]. In addition to its well-known benefits, recent research has highlighted that VD₃ is inextricably linked to gut health in poultry. In a study of yellow-feathered broilers, dietary supplementation with high and medium doses of VD3 (2400 and 3600 IU/kg) was found to improve duodenal intestinal morphology, including increased villus height and decreased crypt depth [22]. Notably, dietary VD3 (25-hydroxyvitamin D3) supplementation has the potential to improve gut health in laying hens infected with coccidiosis, particularly by reducing intestinal permeability and intestinal lesion scores [23]. In vitro studies have revealed that 1.25-dihydroxyvitamin D3 stimulates avian β-defensin expression in the intestinal epithelium of the chicken embryo [24], which is expected to control the disease by enhancing the innate immunity of the host.

Given that previous studies have found that SB combined with VD3 improves chicken antioxidant capacity and early broiler growth performance [25], and that there may be a combined effect in intestinal development [26], we hypothesized that pre-supplementation of the diet with SB and VD3 could counteract the negative effects of cold stress on broiler growth. Therefore, the aim of this study was to investigate the effects of feeding SB and VD3 on broiler growth performance, immune status, antioxidant capacity, and gut health under cold stress.

## 2. Materials and Methods

### 2.1. Birds, Diet, and Experimental Design

All research animal care procedures were approved by the Institutional Animal Care and Ethics Committee of Nanjing Agricultural University (Protocol PAT2022040). A total of 144 1-day-old male Arbor Acres broiler chicks were purchased from Shuangli Poultry Hatchery, Hai’an, Jiangsu Province. The birds were randomly divided into three groups of 6 replicate cages of 8 chicks per cage for a 21–d experiment. The experimental groupings were as follows: group 1, CON (received basal diet); group 2, CS (received basal diet + cold stress); and group 3, CS+B+VD (received SB/VD3 diet + cold stress). The basal diet (Table S1) was based on corn–soybean meal formulations and provided to the birds from 0 to 21 d of age. For the SB/VD3 diet, the basal diet was supplemented with 2000 IU/kg VD3 (Jusuo Bio Technology Co., Ltd., Henan, China) and 1 g/kg SB (30%, Hangzhou King Techina Feed Co., Ltd., Hangzhou, Zhejiang, China). All diets were formulated to meet or exceed the NRC (1994) [27] nutrient recommendations. On d 18, all the CS birds were submitted to acute cold stress for 72 h [3] at a temperature of 15 ± 1 °C, and the CON group was kept at a normal temperature of 26 ± 1 °C. All birds had ad libitum access to feed and water throughout the experiment. The photoperiod was set to 24 h of light for the first three days and adjusted to an 18:6 period of light and dark for the rest of the trial.

### 2.2. Growth Performance

The body weight of each replicate and the weight of the remaining feed of each replicate were recorded at 8 AM on d 18. Then, the broiler chickens were subjected to different environment temperatures. At 8 AM on d 21, the BW of each replicate was recorded, and the weight of the remaining feed of each replicate was recorded. The average BW gain, average feed intake, and feed conversion ratio (FCR) were calculated for the period of 1–18 d, 18–21 d and 1–21 d (*n* = 6).

### 2.3. Sample Collection

After recording the body weight and feed weight, one bird was randomly selected from each replicate (*n* = 6). About 5 mL of blood was drawn from the brachial vein, and serum was separated through centrifugation at 3000 rpm for 10 min at 4 °C and then stored at −20 °C. The broiler chickens were euthanized by exsanguination after electrical stunning. The thymus, spleen, bursa of Fabricius, cecal tonsils, and intestine were removed separately and weighed by trained individuals for calculation of the organ index using the following equation: organ index (%) = weight of organs (g)/live chicken weight (g) × 100. Approximately 2 cm of the ileum was carefully removed and placed in 4% paraformaldehyde for histologic analysis. Furthermore, ileum, ileal mucosa, and ileal content samples were collected in RNase- and DNase-free tubes and immediately snap-frozen in liquid nitrogen, then stored at −80 °C for further analysis.

### 2.4. Histological Analyses

The fixed ileum tissues from 6 birds per group were longitudinally cross-sectioned and subsequently embedded in paraffin wax. After sectioning the tissues into 5 μm slices via a microtome along the cross-section, they were stained with Alcian blue-PAS for counting analysis of goblet cells. Image capture and photography were executed using a digital camera (DS-U3, Nikon, Japan). Goblet cell quantification entailed the examination of five villi from each section, along with measurement of villus epithelium length. Subsequently, the number of goblet cells per unit length (mm) was computed. Furthermore, two additional consecutive sections from the ileum were subjected to immunohistochemical analyses for cluster of differentiation 3 (CD3) and proliferating cell nuclear antigen (PCNA). Morphometric analysis was conducted with Image-Pro Plus 6.0 software (Media Cybernetics, Inc., Rockville, MD, USA). For each ileum segment, a total of 12 well-oriented and intact villi as well as 12 crypts, selected from 3–4 photomicrographs of one slide, were employed for the evaluation of villus height (VH, measured from the villus tip to the crypt), crypt depth (CD, measured from the villus base to the submucosa), and the villus height to crypt depth ratio (VH/CD). Then, following previous protocols, all sections were semi-quantitatively assessed using Image-Pro Plus 6.0 software. The average optical density was derived from the ratio of integrated optical density to the area in three images for each sample.

### 2.5. Antioxidant-Related Biochemical Kit Assay

The prepared ileal homogenates and serum samples were utilized for the assessment of antioxidant-related parameters. The activities of total antioxidant capacity (T-AOC, Catalog No. A015), superoxide dismutase (SOD, Catalog No. A001-1-1), catalase (CAT, Catalog No. A007-1-1), and glutathione peroxidase (GSH-Px, Catalog No. A005-1-2), along with the concentrations of malondialdehyde (MDA, Catalog No. A003-1), were determined through colorimetric assays employing commercially available kits in accordance with the provided manuals (Nanjing Jiancheng Bioengineering Institute, Nanjing, China). Measurements were taken using a microplate reader at specified wavelengths.

### 2.6. Enzyme-Linked Immunosorbent Assay

Chicken-specific enzyme-linked immunosorbent assay (ELISA) kits purchased from AoQing Technology Co., Ltd. (Nanjing, China) were used to determine inflammatory cytokine (IL-1β, ANG-E32218C; IL-2, ANG-E32014C; IL-4, ANG-E32064C; IL-10, ANG-E32011C; IFN-γ, ANG-E32003C; TGF-β, ANG-E32223C) and immunoglobulin (IgG, ANG-E32009C) concentrations in the collected serum samples, as well as indicators of stress (Adrenocorticotropic hormone, ACTH, ANG-E32122C; Corticosterone, CORT, ANG-E32176C), leaky gut (D-lactic acid, D-LA, ANG-E32105C; Endotoxin, ET, ANG-E32160C), and secretory immunoglobulin A (sIgA, ANG-E32006C) in the mucosal samples. All operations were strictly consistent with the kit instructions.

### 2.7. Real-Time Quantitative PCR Analysis

The ileal samples were promptly ground into a fine powder using cryogenic liquid nitrogen. Subsequently, 50 mg of the powdered sample was weighed and transferred to 1 mL of TRIzol reagent (Cat No. R401-01, Vazyme, Nanjing, China) for total RNA extraction. The concentration of the extracted tissue RNA was assessed utilizing an ultra-micro ultraviolet spectrophotometer (NanoDrop2000, ThermoFisher Scientific Co., Ltd., Waltham, MA, USA) in accordance with the manufacturer’s instructions. Following the guidelines provided by the reverse transcription kit (Cat No. R333-01, Vazyme, Nanjing, China), a reverse transcription reaction was employed to convert mRNA into cDNA, which was subsequently stored at −20 °C. Taq Pro Universal SYBR qPCR Master Mix (Cat No. Q712, Vazyme, Nanjing, China) was used for qPCR, performed on an ABI PRISM 7300 HT sequence detection system (Applied Biosystems Inc., Waltham, MA, USA). The primers utilized for qPCR are detailed in Table S2 and were synthesized by Sangon Biotech (Shanghai) Co. Ltd., China. Relative gene expression data were analyzed using the 2^−ΔΔCt^ method.

### 2.8. 16S rRNA Gene Sequencing

Genomic DNA from the ileum samples’ microbial community was extracted using the E.Z.N.A.^®^ soil DNA Kit (Omega Bio-tek, Norcross, GA, USA) following the manufacturer’s instructions. Subsequently, the DNA concentration was determined using a NanoDrop 2000 UV–Vis spectrophotometer (Thermo Fisher Scientific, Waltham, MA, USA), and the quality of the DNA was assessed through 1% agarose gel electrophoresis. The bacterial 16S RNA V3–V4 gene region was amplified via polymerase chain reaction (PCR) employing primers 338F (5′-ACTCCTACGGGAGGCAGCAG-3′) and 806R (5′-GGACTACHVGGGTWTCTAAT-3′). The PCR product was then extracted from a 2% agarose gel and purified utilizing the AxyPrep DNA Gel Extraction Kit (Axygen Biosciences, Union City, CA, USA) according to the manufacturer’s guidelines. The purified product was quantified using a Quantus™ Fluorometer (Promega, San Luis Obispo, CA, USA). Finally, 16S rRNA gene sequencing was carried out using either an Illumina MiSeq PE300 platform or NovaSeq PE250 platform (Illumina, San Diego, CA, USA), following the standard protocols conducted by Sanshu Biotechnology Co., Ltd. (Shanghai, China).

### 2.9. Statistical Analysis

A one-way analysis of variance (ANOVA) was used to analyze the experimental data between groups, with the exception of the microbial part, using SPSS 18.0 statistical software (SPSS Inc., Chicago, IL, USA). Tukey’s post hoc test was used to determine the differences among treatment groups. Regarding the microbial part, the Wilcoxon test was used to examine features with significantly different microbiota abundances. Data are shown as mean values with the standard error of the total mean (SEM). For all tests, *p* < 0.05 was considered a significant difference.

## 3. Results

### 3.1. Growth Performance and Organ Index

At 18 and 21 d of age, we observed reduced ADG and elevated FCR in cold-stressed birds compared to CON birds (*p* < 0.05, Table 1). Notably, birds fed the SB/VD3 diet had higher BW and ADG under cold exposure throughout the 1–21 d experimental period (*p* < 0.05). In addition, both cold stress and the SB/VD3 diet had no effect on the organ index in broiler chicks (*p* > 0.05, Table S3).

### 3.2. Intestinal Morphology and Immuno-Histochemical Analyses

**Figure 1:**
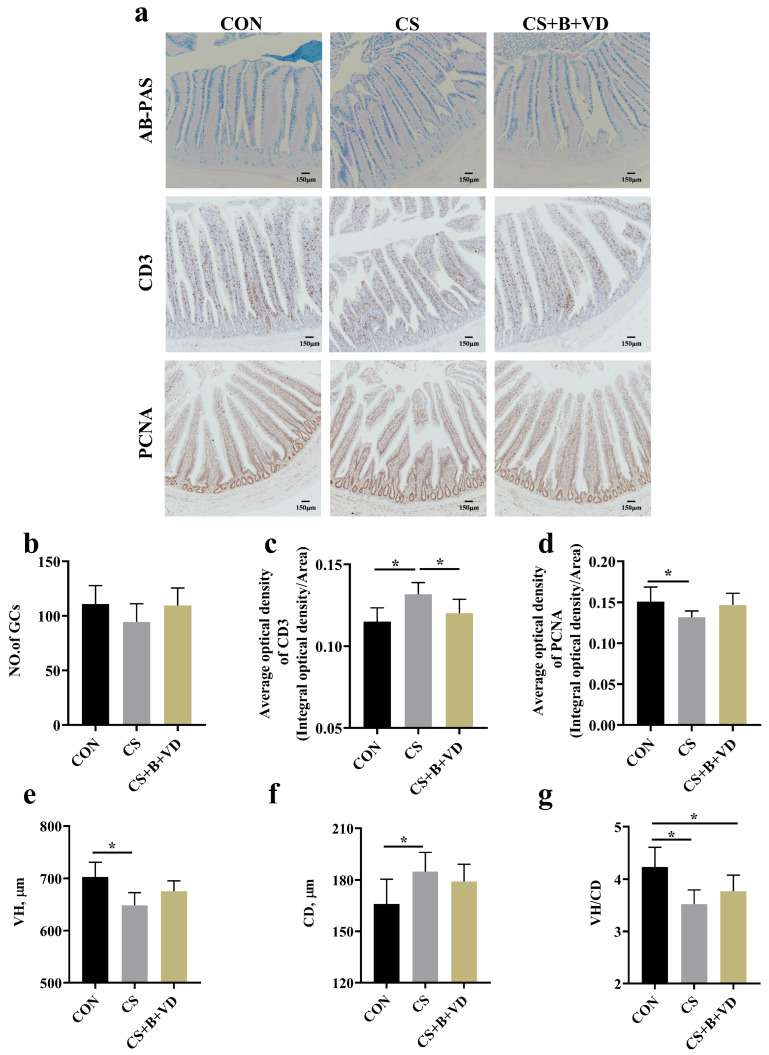
Effects of dietary sodium butyrate and vitamin D3 supplementation on ileal morphology, number of goblet cells, CD3 and PCNA expression of cold stress-challenged birds. Representative AB-PAS staining, CD3 and PCNA immuno-histochemical images (**a**). No. of goblet cells (**b**), CD3 (**c**) and PCNA (**d**) expression. VH (**e**), CD (**f**) and VH/CD (**g**). Data represent mean ± SEM (*n* = 6), and differences were analyzed by a one-way ANOVA following by Tukey’s multiple-range test among three groups, * *p* < 0.05. Abbreviations: CON, non-challenged birds fed a basal diet; CS, cold-stressed birds fed a basal diet; CS+B+VD, cold-stressed birds fed a basal diet supplemented with 1 g/kg sodium butyrate and 2000 IU/kg vitamin D3. GCs, goblet cells; VH, villus height; CD, crypt depth; VH/CD, ratio of villus height to crypt depth; CD3, cluster of differentiation 3; PCNA, proliferating cell nuclear antigen.

Compared with CON birds, cold stress increased the expression of CD3 (Figure 1c) and decreased the expression of PCNA in the ileum of birds (*p* < 0.05, Figure 1d), while the expression of CD3 was reduced (*p* < 0.05) by SB/VD3 supplementation. Meanwhile, CS birds had lower VH and VH/CD and higher CD compared to the CON birds (*p* < 0.05).

### 3.3. Antioxidant Parameters

**Figure 2:**
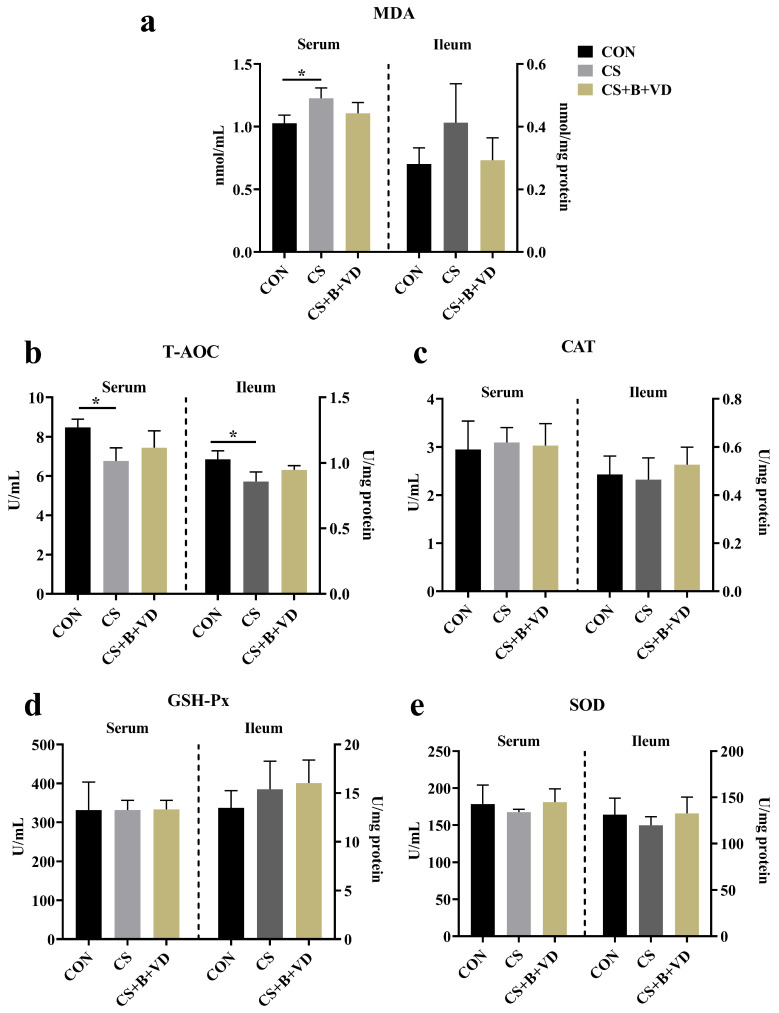
Effects of dietary sodium butyrate and vitamin D3 supplementation on the antioxidant capacity of cold stress-challenged birds. MDA (**a**), T-AOC (**b**), CAT (**c**), GSH-Px (**d**) and SOD (**e**), as described in the materials and methods section. Data represent mean ± SEM (*n* = 6), and differences were analyzed by a one-way ANOVA following by Tukey’s multiple-range test among three groups, * *p* < 0.05. Abbreviations: CON, non-challenged birds fed a basal diet; CS, cold-stressed birds fed a basal diet; CS+B+VD, cold-stressed birds fed a basal diet supplemented with 1 g/kg sodium butyrate and 2000 IU/kg vitamin D3.

Compared with CON birds, CS birds exhibited higher accumulation of MDA in the serum (*p* < 0.05, Figure 2a). Additionally, cold stress decreased T-AOC activity in the serum and ileum (*p* < 0.05, Figure 2b).

### 3.4. Immunity, Stress-Related Hormones, and Gut Barrier Biomarkers

**Figure 3:**
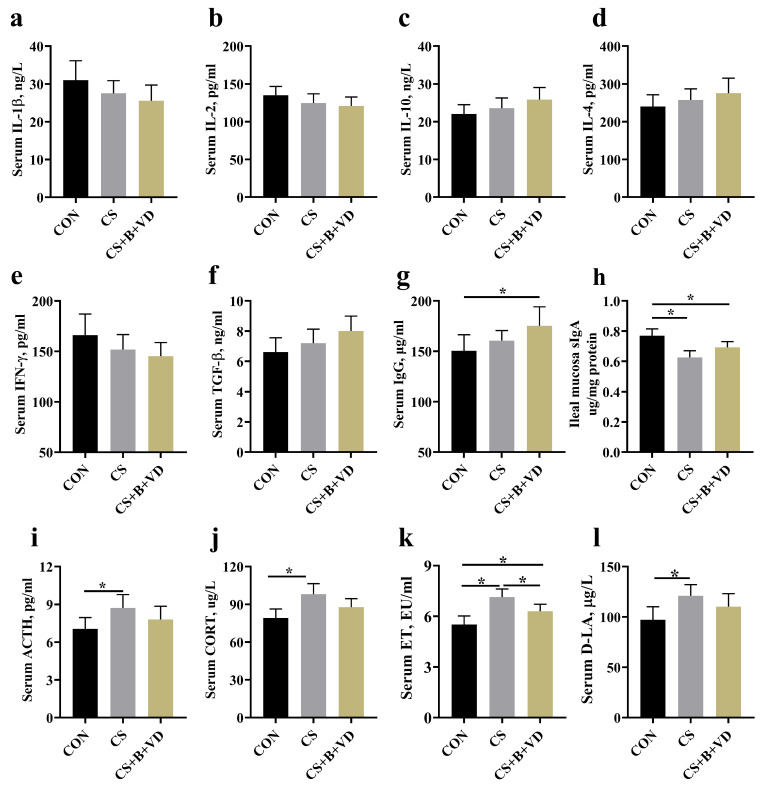
Effects of dietary sodium butyrate and vitamin D3 supplementation on inflammatory factors, immunoglobulins, stress-related hormones, and leaky gut biomarkers of cold stress-challenged birds. IL-1β (**a**), IL-2 (**b**), IL-10 (**c**), IL-4 (**d**), IFN-γ (**e**), TGF-β (**f**), IgG (**g**), sIgA (**h**), ACTH (**i**), CORT (**j**), ET (**k**) and D-LA (**l**), as described in the materials and methods section. Data represent mean ± SEM (*n* = 6), and differences were analyzed by a one-way ANOVA following by Tukey’s multiple-range test among three groups, * *p* < 0.05. Abbreviations: CON, non-challenged birds fed a basal diet; CS, cold-stressed birds fed a basal diet; CS+B+VD, cold-stressed birds fed a basal diet supplemented with 1 g/kg sodium butyrate and 2000 IU/kg vitamin D3.

In comparison with birds from the CON, CS+B+VD birds exhibited a higher IgG level in the serum (*p* < 0.05, Figure 3g). CS and CS+B+VD birds possessed lower ileal mucosal sIgA levels than CS birds (*p* < 0.05, Figure 3h). Cold stress elevated the concentrations of ACTH (Figure 3i), CORT (Figure 3j), and D-LA (Figure 3l) in the serum when compared with the CON group (*p* < 0.05). Serum ET levels were elevated in CS and CS+B+VD birds compared to CON birds (*p* < 0.05). However, the SB/VD3 diet reduced serum ET levels compared to CS birds (*p* < 0.05, Figure 3k).

### 3.5. Ileal Gene Expression

**Figure 4:**
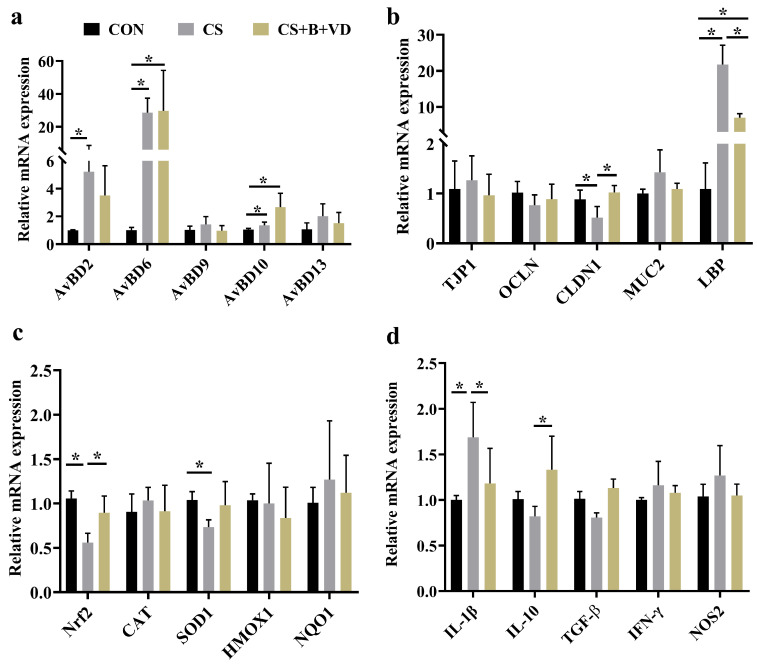
Effects of dietary sodium butyrate and vitamin D3 supplementation on mRNA abundance of ileal genes of cold stress-challenged birds. The levels of defensin (**a**), barrier (**b**), oxidative stress (**c**) and inflammation (**d**) related genes in the ileum. Data represent mean ± SEM (*n* = 6), and differences were analyzed by a one-way ANOVA following by Tukey’s multiple-range test among three groups, * *p* < 0.05. Abbreviations: CON, non-challenged birds fed a basal diet; CS, cold-stressed birds fed a basal diet; CS+B+VD, cold-stressed birds fed a basal diet supplemented with 1 g/kg sodium butyrate and 2000 IU/kg vitamin D3. AvBDs, Avian beta-defensins; Nrf2, nuclear factor erythroid 2-related factor 2; CAT, catalase; SOD1, superoxide dismutase 1; HMOx1, heme oxygenase 1; NQO1, NAD(P)H quinone dehydrogenase 1; TPJ1, tight junction protein 1; OCLN, occludin; CLDN1, claudin 1; MUC2, mucin 2; LBP, lipopolysaccharide binding protein; IL-1β, interleukin-1β; IL-10, interleukin-10; TGF-β, transforming growth factor beta; IFN-γ, interferon gamma; NOS2, nitric oxide synthase 2; GAPDH, glyceraldehyde-3-phosphate dehydrogenase.

Cold stress upregulated the mRNA expression of AvBD2, AvBD6, and AvBD10 in the ileum of birds compared to CON birds (*p* < 0.05, Figure 4a). Cold stress downregulated the mRNA expression of nuclear factor erythroid 2-related factor 2 (Nrf2) and claudin 1 (CLDN1) in the ileum compared to CON birds (*p* < 0.05). In contrast, the SB/VD3 diet enhanced the Nrf2 and CLDN1 mRNA expression compared to CS birds (*p* < 0.05, Figure 4b and c). The ileal LBP mRNA expression was elevated in CS and CS+B+VD birds compared to CON birds (*p* < 0.05). However, the SB/VD3 diet decreased LBP mRNA expression compared to CS birds (*p* < 0.05, Figure 4b). Cold stress decreased ileal superoxide dismutase 1 (SOD1) mRNA expression compared to CON birds (*p* < 0.05, Figure 4c), whereas the SB/VD3 diet increased ileal IL-10 mRNA expression compared to CS birds (*p* < 0.05, Figure 4d). Additionally, in comparison with the CS birds, CS+B+VD birds exhibited higher mRNA expression of IL-10 in the ileum (*p* < 0.05, Figure 4d).

### 3.6. Ileal Microbial Profile

**Figure 5:**
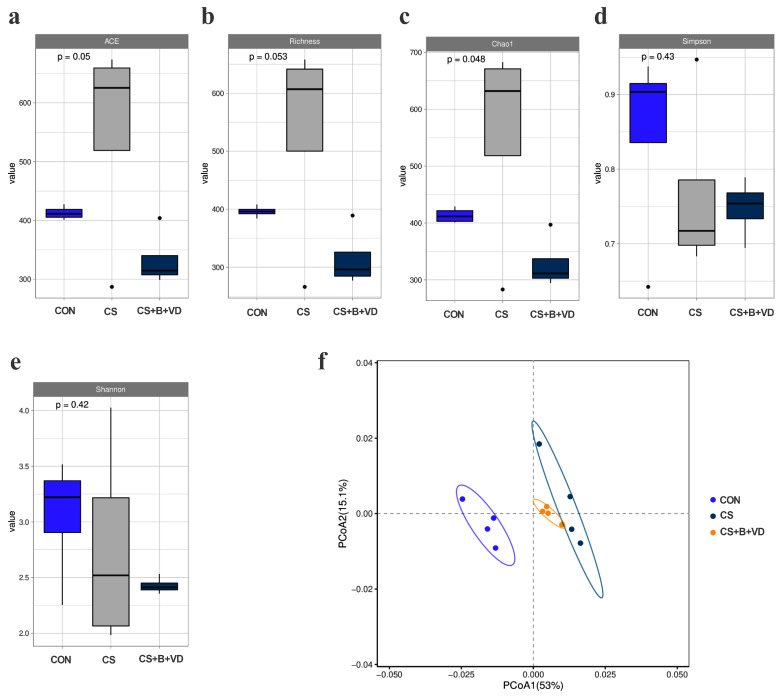
Effects of dietary sodium butyrate and vitamin D3 supplementation on the diversity analysis of ileal microbiota of cold stress-challenged birds. (**a**–**e**) Alpha indices of the ileal microbiota from the three groups. (**f**) Ileal microbiota structure of broiler in the three groups by PCoA score plots based on weighted UniFrac distance. Data are expressed as box plots (*n* = 4). Abbreviations: CON, non-challenged birds fed a basal diet; CS, cold-stressed birds fed a basal diet; CS+B+VD, cold-stressed birds fed a basal diet supplemented with 1 g/kg sodium butyrate and 2000 IU/kg vitamin D3.

**Figure 6:**
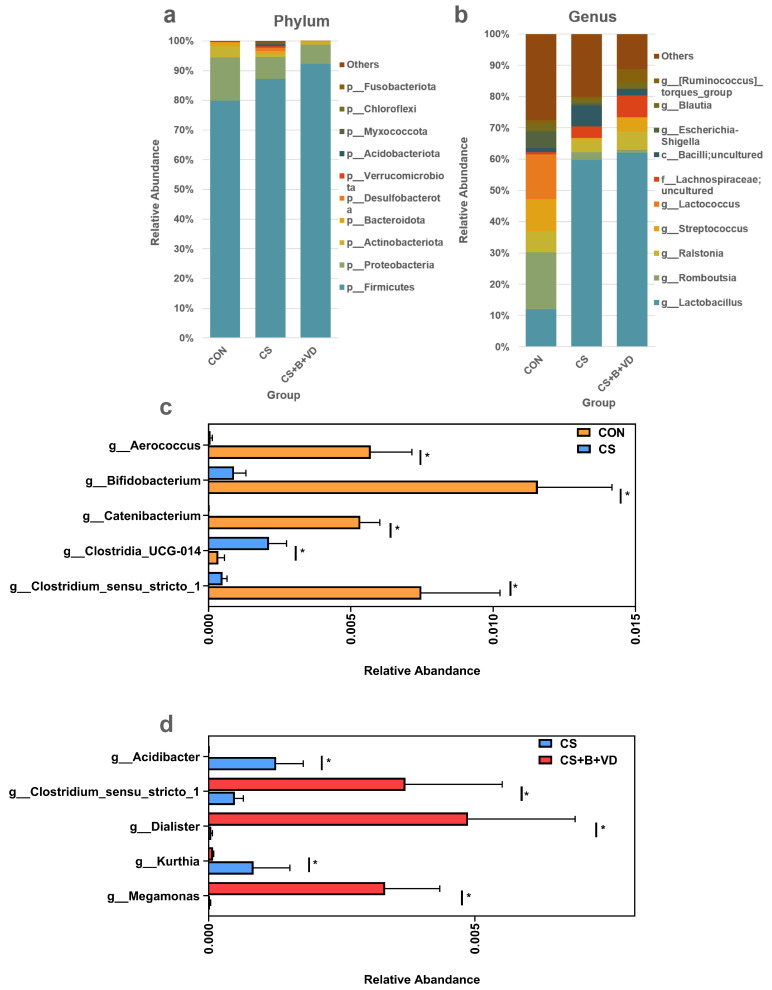
Effects of dietary sodium butyrate and vitamin D3 supplementation on the ileal microbial composition of cold stress-challenged birds. Top ten gut microbial compositions at the phylum (**a**) and genus (**b**) levels. Differential microbiota at the genus level ((**c**), CON vs CS; (**d**), CS vs CS+B+VD). Data are expressed as bar plots (*n* = 4), and differences were analyzed by the Wilcoxon test, * *p* < 0.05. Abbreviations: CON, non-challenged birds fed a basal diet; CS, cold-stressed birds fed a basal diet; CS+B+VD, cold-stressed birds fed a basal diet supplemented with 1 g/kg sodium butyrate and 2000 IU/kg vitamin D3.

The chao1 index of CS birds was the highest (*p* < 0.05, Figure 5c). PCoA showed a significant separation of ileal microbial communities between three groups (Figure 5f). At both the phylum and genus levels, birds in the CS and CS+B+VD group showed distinct gut bacterial compositions compared with those of CON birds (Figure 6a,b). At the genus level, cold stress decreased (*p* < 0.05) the relative abundance of *Clostridium_sensu_stricto_1*, *Catenibacterium*, *Bifidobacterium*, and *Aerococcus* and increased the relative abundance of *Clostridia_UCG-014* in the broiler ileum, compared with the CON group (Figure 6c). However, a significant increase in the relative abundance of *Megamonas*, *Dialister* and *Clostridium_sensu_stricto_1* and a significant decrease (*p* < 0.05) in *Kurthia* and *Acidibacter* were observed in the ileum of CS broilers receiving the SB/VD3 diet compared to the CS birds (Figure 6d).

## 4. Discussion

In the present experiments, birds under acute cold stress showed poor growth performance parameters, including reduced ADG and increased FCR. However, the SB/VD3 diet restored the cold-induced reduction in ADG. The reduction in growth performance due to cold stress can be primarily attributed to the following factors. Cold stress elevates basal metabolic rate and energy metabolism [28], necessitating continuous energy expenditure to maintain body temperature. This results in the redirection of nutrients from growth promotion towards thermoregulation, leading to a decrease in body weight gain in broilers. Cold stress initiates the activation of the hypothalamo–pituitary–adrenocortical response axis [29], leading to increased levels of ACTH and CORT. This aligns with the findings of the current experiment. This heightened hormonal response augments gluconeogenesis while hindering body weight growth in broilers [30]. Additionally, cold stress can disrupt intestinal homeostasis [31], potentially impacting the digestion and absorption of nutrients. This study primarily assessed the adverse effects of cold stress on broiler chicks’ performance from the perspective of gut health, while also investigating the protective effects of combined supplementation with the SB/VD3 diet.

Apart from its direct adverse effects on growth performance, cold stress disrupts internal homeostasis, posing a substantial threat to intestinal health. Previous reports have noted that cold temperatures can lead to pathological damage, such as congestion and necrosis, in the intestinal mucosa of broilers [4]. Some studies have even indicated that cold exposure heightens the incidence of necrotizing enteritis in broiler chicks [6]. Nevertheless, in this experiment, no significant pathological changes were observed in the intestinal mucosa resulting from cold stress. Only villi atrophy and a decreased proliferation rate of intestinal epithelial cells were detected. Pre-supplementation of diets with SB/VD3 did not fully mitigate these adverse effects, yielding only a modest improvement. It is worth noting that this observed trend of improvement may ultimately be attributed to the direct energy-supplying effect of butyrate on intestinal epithelial cells [32]. A study found that increased concentrations of butyrate in the intestinal lumen resulted in accelerated proliferation of small intestinal crypt cells [33]. The intricate interplay between VD3 and SB on cell proliferation and intestinal villus development requires further elucidation through subsequent studies.

Changes in intestinal oxidative status often coincide with shifts in intestinal morphology and permeability [34]. Initially, this study observed that cold stress led to an augmented accumulation of the lipid peroxidation product MDA, accompanied by a decrease in total antioxidant capacity. This observation underscored that cold stress disrupted the dynamic equilibrium between oxygen radical reactions and lipid peroxidation reactions in broiler chickens, resulting in excessive production of free radicals in the body and inducing oxidative damage. This phenomenon was indirectly confirmed by the reduced mRNA expression levels of ileal Nrf2 and SOD1. In normal physiological conditions, when the organism encounters oxidative stress due to an excess of reactive oxygen species, the antioxidant system is triggered. Activated Nrf2 then translocates into the nucleus to mitigate oxidative damage [35]. While appropriate cold stress may promote Nrf2 activation, prolonged or acute cold stress could potentially lead to compromised or absent Nrf2 activation [36]. Generally, an imbalance in the organism’s redox system can result in biofilm damage, subsequently compromising tissue integrity. Unsurprisingly, traditional markers of leaky gut in the serum including D-LA and ET exhibited elevated levels, indicating heightened intestinal permeability. This observation is in accordance with alterations in the mRNA expression of LBP in the ileum. At the gene expression level, the cold stress-induced decrease in the expression of CLDN1, a small membrane-penetrating protein that constitutes a tight junction, was completely reversed by the SB/VD3 diet. This observation may be mechanistically explained by the role of butyrate as a histone deacetylase (HDAC) inhibitor, which impedes the inhibition of CLDN1 transcription by HDAC [37].

Changes in inflammatory response and immune regulatory capacity are likewise key considerations in broilers exposed to cold. Several studies have suggested that the translocation of endotoxins from the intestinal tract to the systemic circulation, facilitated by increased intestinal permeability, may be a contributing factor to the development of endotoxemia [38]. This condition is typically accompanied by shifts in inflammatory markers. Unexpectedly, this study found no alterations in the levels of either pro- or anti-inflammatory cytokines in the serum of cold-exposed birds. The only discernible change was an increase in the mRNA abundance of intestinal IL-1β. Additionally, the expression of CD3, a surface marker representing the number of intestinal T lymphocytes [39], was significantly increased. Typically, the quantity of intestinal lymphocytes corresponds to the intensity of the inflammatory response [40]. The observed discrepancy between changes in systemic and localized inflammatory responses may be attributed to the duration of cold exposure. Inclusion of the SB/VD3 diet reduced the intestinal inflammatory response in cold-stressed broilers, possibly due to the potent anti-inflammatory activity of SB and VD3 [41,42]. In addition, CS birds exhibited a notably attenuated sIgA level. As the major immunoglobulin in the intestinal mucosa, sIgA is the first line of defense in the intestinal mucosa against a variety of endogenous commensal bacteria and exogenous invading pathogens. Decreased sIgA levels implied that cold dulled the mucosal immune response, which may be related to B-cell activity; plasma cell differentiation and proliferation; and the number of intestinal commensal bacteria [43]. Notably, the expression of intestinal avian defensin was significantly upregulated in response to cold exposure, regardless of whether supplementation with SB/VD3 was provided. Recent studies have noted that intermittent cold stimulation decreased AvBDs expression in broiler thymus and bursa [29], which is inconsistent with the current study. This inconsistency is not unexpected because the activation of intestinal defensins is more dependent on the increase in lipopolysaccharide (LPS) and bacterial metabolites due to leaky gut, while the effect of cold on intestinal permeability is obvious.

The dynamic balance of the gut microbiota is critical for animal health and growth and is influenced by many factors, including environmental stress and diet. Cold stress has been reported to alter the composition of the gut microbiota, thereby triggering colitis [44]. Changes in the composition and diversity of broiler gut microbiota due to cold were observed in the current experiment. Specifically, cold increased the relative abundance of *Clostridia_UCG-014* and decreased *Clostridium_sensu_stricto_1* and *Bifidobacterium*. *Clostridia_UCG-014* may be a potential pro-inflammatory bacterium with increased abundance in the rumen of cows with subclinical mastitis [45]. *Clostridium_sensu_stricto_1* emerges as the predominant colony in chick feces post-birth [46], and it is widespread in the environment. This bacterium is generally linked to conditions like necrotizing enteritis [47] and stress [48], and it plays a significant role in the production of short-chain fatty acids [49]. Notably, the abundance of *Clostridium_sensu_stricto_1* increased with SB/VD3 supplementation. Further research is necessary to elucidate its physiological impacts on broiler growth and associated traits. Furthermore, the relative abundance of the recognized probiotic *Bifidobacterium* significantly decreased in the CS birds. Conversely, *Megamonas* showed higher prevalence in broiler guts following the SB/VD3 diet. *Megamonas* has been linked to the development of the immune system in broilers [50]. Overall, cold stress has a negative impact on broiler gut microbiota homeostasis, while the SB/VD3 diet has potential countermeasures.

## 5. Conclusions

In conclusion, the early SB/VD3 diet effectively alleviated the adverse impacts of cold stress on broiler growth performance and gut health. This was achieved through improvements in reinforced intestinal barrier function and anti-inflammatory capacities and the stabilization of gut microbiota balance in broiler chicks. These results are useful in helping farmers to manage cold stress in broiler farming. Additionally, the results also provide novel insights into the further investigation of the effects of SB/VD3 diet on the production, health, and welfare of broiler chickens undergoing cold transportation or other stressful conditions, such as crowding stress, heat stress, and immunization stress.

## Figures and Tables

**Table 1:** Effect of supplementation with sodium butyrate and vitamin D3 on growth performance of cold-stressed broiler chicks.

Items ^1^	CON	CS	CS+B+VD	SEM ^2^	*p*-Value
BW (g)					
18 d	516.00	525.06	526.77	2.52	0.101
21 d	650.17 ^a^	625.03 ^b^	645.90 ^a^	4.39	0.032
ADG (g/d)					
1–18 d	26.27	26.77	26.87	0.14	0.101
18–21 d	44.45 ^a^	33.06 ^b^	39.45 ^ab^	1.67	0.010
1–21d	28.87 ^a^	27.67 ^b^	28.66 ^a^	0.21	0.032
ADFI (g/d)					
1–18 d	30.93	31.33	30.29	0.25	0.111
18–21 d	68.88	65.00	68.42	0.91	0.165
1–21d	36.36	36.14	35.74	0.30	0.714
FCR (g/g)					
1–18 d	1.18	1.17	1.13	0.01	0.064
18–21 d	1.58 ^b^	1.99 ^a^	1.75 ^ab^	0.07	0.044
1–21d	1.26	1.31	1.25	0.01	0.121

^1^ ADFI, average daily feed intake; ADG, average daily gain; BW, body weight; FCR, feed conversion ratio; Abbreviations: CON, non-challenged birds fed a basal diet; CS, cold-stressed birds fed a basal diet; CS+B+VD, cold-stressed birds fed a basal diet supplemented with 1 g/kg sodium butyrate and 2000 IU/kg vitamin D3. ^2^ SEM, standard error of the mean (*n* = 6). ^a–b^ Means within a row with different superscripts are different at *p* < 0.05.

## Data Availability

The original contributions presented in this study are included in the article/Supplementary Materials. Further inquiries can be directed to the corresponding author.

## Supplementary Materials

The following supporting information can be downloaded at: https://www.mdpi.com/article/10.3390/ani15060861/s1, Table S1: Composition and nutrient levels of the basal diet (%, air-dry basis) used in this study for broiler chicks (1–21 d); Table S2 Gene-specific primers of related genes for broilers (Gallus gallus domesticus); Table S3 Effect of supplementation with sodium butyrate and vitamin D3 on organ index of cold-stressed broiler chicks.
