# Supplementary material for: Growth Performance and Gut Health of Cold-Stressed Broilers in Response to Supplementation with a Combination of Sodium Butyrate and Vitamin D3
Source: Animals (Basel). 2025 Mar 17;15(6):861. doi: 10.3390/ani15060861 (PMC11939318; doi:10.3390/ani15060861)
Supplement: Supplementary file 1 [file animals-15-00861-s001.zip › animals-3509500-supplementary.pdf]

Table S1 Composition and nutrient levels of the basal diet (%, air-dry basis) used in this study for broiler chicks (1-21 d).

Items	Content, %
Corn	57.0
Soybean meal	30.0
Corn gluten meal	4.00
Soybean oil	4.00
Limestone	1.20
Dicalcium phosphate	2.05
L-Lysine	0.24
DL-Methionine	0.21
Sodium chloride	0.30
Premix[2]	1.00
Total	100
Calculated nutrient levels, %	
Apparent metabolizable energy (Mcal/kg)	3.04
Crude protein	20.44
Calcium	1.00
Total phosphorus	0.73
Available phosphorus	0.45
Lysine	1.16
Methionine	0.54
Methionine + cystine	0.87
Analyzed nutrient levels, %	
Crude protein	20.9
Calcium	1.08
Total phosphorus	0.80
Lysine	1.06
Methionine	0.56
Methionine + cystine	0.88

[1]Premix provided per kilogram of diet): Vitamin A (Transretinyl acetate), 10,000 IU; Vitamin E (all-rac-α-tocopherol), 30 IU; Vitamin D3, 3000 IU; Menadione, 1.3 mg; Thiamin, 2.2 mg; Riboflavin, 8 mg; Nicotinamide, 40 mg; Choline chloride, 600 mg; Calcium pantothenate, 10 mg; Pyridoxine·HCl, 4 mg; Biotin, 0.04 mg; Folic acid, 1 mg; Vitamin B_{12} (Cobalamin), 0.013 mg; Fe (from ferrous sulfate), 80 mg; Cu (from copper sulphate), 8.0 mg; Mn (from manganese sulphate), 110 mg; Zn (from zinc oxide), 60 mg; I (from calcium iodate), 1.1 mg; Se (from sodium selenite), 0.3 mg

Table S2 Gene-specific primers of related genes for broilers (Gallus gallus domesticus).

Gene[1]	Accession number	Primer pairs, 5′ → 3′	Product length
AvBD2	XM_046913156.1	F: CTCTCCTCTTCCTGGCACTC<br>R: TCCAAAGTGGCAGGACCCTC	92
AvBD6	NM_001001193.1	F: TCTGTCCTCTTTGTGGTGCTC<br>R: CACTGCCACATGATCCAACCC	142
AvBD9	NM_001001611.3	F: AGCCAAGAAGATGCTGACACC<br>R: CCCAATGTCAACTGAAGGAGCAC	90
AvBD10	NM_001001609.3	F: AGATCCTCTGCCTGCTCTTCG<br>R: CCCAGCACGGCAGAAATTCCC	122
AvBD13	NM_001001780.2	F: AGCTGTGCAGGAACAACCAT<br>R: GCCGTTCATGCAGCTCCCA	80
NRF2	NM_001396902.1	F: AGAATGACAAAAGCCTTCG<br>R: CATCTTCATCACGCAGCAT	84
CAT	NM_001031215.2	F: ACGCTCAACTTTTCATTCAGA<br>R: AGCATTGTATTTGTCCAGCAG	101
SOD1	NM_205064.2	F: CACACTGCATCATTGGCCGTA<br>R: CCACAAGCTAAACGAGGTCCA	115
HMOX1	NM_205344.2	F: GCCACCAAGTTCAAACAGCTC<br>R: AATGCTTTCTTGGCTTCCTC	98
NQO1	NM_001277619.2	F: AAGCCATGCTGTCACTCACCAC<br>R: CAGCACGTTCATGTCGCCGTT	80
TJP1	XM_040706827.2	F: CAAGAGCCATCATCCTTACCG<br>R: TGTTGACTGCTCGTACTCCCT	216
OCLN	NM_205128.1	F: CGGCAGCACCTACCTCAACCAGT<br>R: GGAACCCACAGACAGCAGCCACA	83
CLDN1	NM_001013611.2	F: CCGCCATACTCCTGGGTCT<br>R: CCATCCGCATCTTCTTCACCT	99
MUC2	XM_040701656.2	F: AAAATGTATCTGTCGCCCCTC<br>R: GTCGCCATCCTTTATTGTTGC	121
LBP	XM_004947186.5	F: CTGGCCCCTCTATTCCTCC<br>R: GAGAGCTTCATCCTGCCCACA	107
IL-1β	XM_015297469.3	F: GTCAACATCGCCACCTACAA<br>R: AACGGGACGGTAATGAAACA	193
IL-10	NM_001004414.4	F: CCATGCAGACCAGCACCAG<br>R: TCTGCTTGATGGCTTTGCTCCT	132
TGF-β	NM_205454.2	F: TGCCCTTGCCATACTTTTCAGC<br>R: GTCCCCACGGCCATAGTCA	111
IFN-γ	NM_205427.1	F: TACTGAGCCAGATTGTTTCGAT<br>R: TTTCACCTTCTTCACGCCAT	134
NOS2	NM_204961.2	F: CTCTTTGCGTCATTACTCCTG<br>R: TTTCCCAGTCTCGGTTGCAT	81
GAPDH	NM_204305.2	F: GAACATCATCCCAGCGTCCA<br>R: CGGCAGGTCAGGTCAACAAC	132

[1]AvBDs, Avian beta-defensins; NRF2, nuclear factor erythroid 2-related factor 2; CAT, catalase; SOD1, superoxide dismutase 1; HMOx1, heme oxygenase 1; NQO1, NAD(P)H quinone dehydrogenase 1; TPJ1, tight junction protein 1; OCLN, occludin; CLDN1, claudin 1; MUC2, mucin 2; LBP, lipopolysaccharide binding protein; IL-1β, interleukin-1β; IL-10, interleukin-10; TGF-β, transforming growth factor beta; IFN-γ, interferon gamma; NOS2, nitric oxide synthase 2; GAPDH, glyceraldehyde-3-phosphate dehydrogenase

Table S3 Effect of supplementation with sodium butyrate and vitamin D3 on organ index of cold-stressed broiler chicks.

Organs index[1], %	CON	CS	CS+B+VD	SEM[2]	*P*-value
Thymus	0.28	0.23	0.24	0.01	0.170
Spleen	0.79	0.74	0.71	0.00	0.706
Cecum tonsil	0.03	0.02	0.03	0.00	0.324
Bursa of Fabricius	0.30	0.30	0.28	0.01	0.697
Intestine	4.59	4.71	4.73	0.15	0.931

[1]All the values are calculated relative to the final body weight of the birds

[2]SEM, standard error of the mean (n = 6)

Abbreviations: CON, nonchallenged birds fed a basal diet; CS, cold-stressed birds fed a basal diet; CS+B+VD, cold-stressed birds fed a basal diet supplemented with 1 g/kg sodium butyrate and 2,000 IU/kg vitamin D3
